# Evidence of West Nile virus infection in Nepal

**DOI:** 10.1186/s12879-014-0606-0

**Published:** 2014-11-27

**Authors:** Wiriya Rutvisuttinunt, Piyawan Chinnawirotpisan, Chonticha Klungthong, Sanjaya Kumar Shrestha, Amod Bahadur Thapa, Arjun Pant, Samuel L Yingst, In-Kyu Yoon, Stefan Fernandez, Julie A Pavlin

**Affiliations:** Armed Forces Research Institute of Medical Sciences, Bangkok, Thailand; Walter Reed/AFRIMS Research Unit Nepal, Kathmandu, Nepal; Bharatpur Hospital, Chitwan, Nepal; Sukra Raj Tropical Infectious Diseases Hospital, Kathmandu, Nepal; Armed Forces Health Surveillance Center, Silver Spring, Maryland USA

**Keywords:** West nile virus, Nepal, Febrile illness, Deep sequencing, NGS, Phylogeny

## Abstract

**Background:**

Acute febrile illness is common among those seeking medical care and is frequently treated empirically with the underlying illness remaining undiagnosed in resource-poor countries. A febrile illness study was conducted 2009-2010 to identify known and unknown pathogens circulating in Nepal.

**Method:**

Study methods included diagnostic testing and preliminary ELISA screening of acute and convalescent samples for diseases both known and unknown to be circulating in Nepal, including West Nile virus (WNV). The molecular assays including Polymerase Chain Reaction (PCR), Sanger sequencing and ultra deep sequencing on MiSeq Illumina Platform were conducted to further confirm the presence of WNV.

**Results:**

The study enrolled 2,046 patients presenting undifferentiated febrile illness with unknown etiology. Sera from 14 out of 2,046 patients were tested positive for west nile virus (WNV) by nested Reverse Transcription-Polymerase Chain Reaction (RT-PCR). Only two out of 14 cases were confirmed for the presence of WNV by sequencing and identified as WNV lineage 1 phylogentically. The two patients were adult males with fever and no neurological symptoms from Kathmandu and Bharatpur, Nepal.

**Conclusion:**

Two out of 2,046 serum samples contained fragments of WNV genome resembling WNV lineage 1, which is evidence of the continued spread of WNV which should be considered a possible illness cause in Nepal.

**Electronic supplementary material:**

The online version of this article (doi:10.1186/s12879-014-0606-0) contains supplementary material, which is available to authorized users.

## Background

West Nile virus (WNV) is one of 70 members of the *Flavivirus* genus which are serologically characterized by eight antigenic complexes and nine serotypes [1]. WNV can cause neuroinvasive disease and febrile illnesses resulting in substantial morbidity and mortality in humans and other vertebrates.

WNV is classified phylogenetically into eight different lineages with two main lineages causing outbreaks in humans. Lineage 1 is mostly found in India, Australia, the Middle East, Europe and North America, while lineage 2 is mainly found in sub-Saharan Africa, Europe and the island of Madagascar. Sub-lineages III, IV, V, VI and VIII from lineage 1 are further segregated into distinct regions [2]. WNV has been reported in East Asia including India and China [3],[4]. Although no human cases have been reported, the WNV-carrying Culex mosquito vector can be found in Nepal [5]. In 2006, Pant *et al*. reported neutralizing antibodies against Kunjin virus in porcine sera in Nepal, which is a subtype of WNV [6].

Here, we report the presence of WNV RNA by nested Reverse Transcription-Polymerase Chain Reaction (nested RT-PCR) and further subtyped by deep sequencing from the acute sera of two febrile individuals, without neurological symptoms enrolled in a febrile illness study conducted in Nepal from May 2009 to December 2010.

## Methods

**Figure 1:**
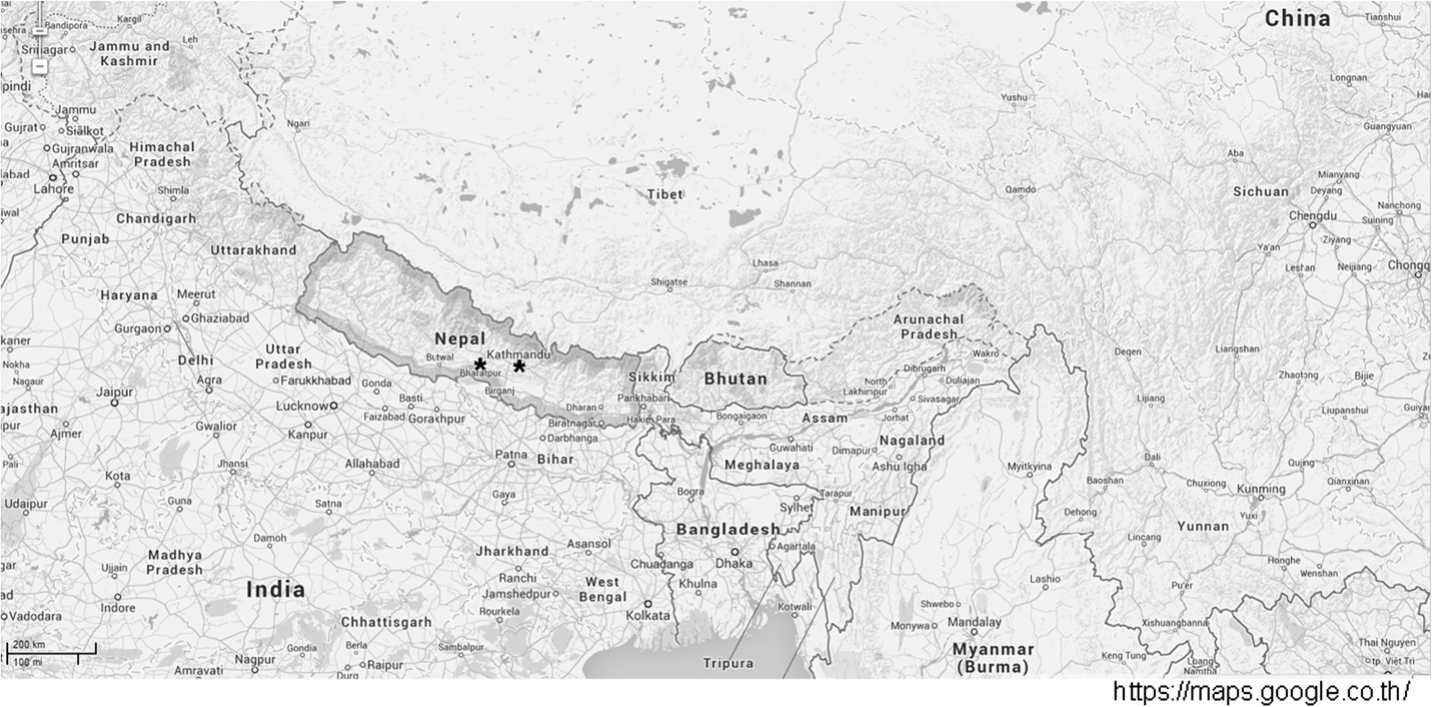
**Map of Kathmandu and Bharatpur cities in Nepal, where the two patients were enrolled during May 2009 and December 2010 for the febrile illness study.**

A total of 2,046 clinical specimens were collected from the febrile illness study at 4 hospitals in 3 cities in Nepal during May 2009 to December 2010. The patients presented with undifferentiated febrile illness with no known etiology. Fourteen acute specimens, from patients whose convalescent samples were WNV antibody positive by Panbio ^(R)^ West Nile Virus IgM Capture ELISA (Alere, GA, USA) (which can have 12.5% cross-reactivity with dengue), were tested by RT-PCR to detect WNV RNA. These serum specimens were collected from febrile volunteers in Kathmandu and Bharatpur (Figure 1) and tested for WNV using a nested RT-PCR adapted from methods previously reported [7],[8]. The febrile illness study was approved by the institutional review boards of the Nepal Health Research Council and Walter Reed Army Institute of Research under WRAIR protocol # 1513, and was performed in compliance with the Helsinki Declaration.

**Figure 2:**
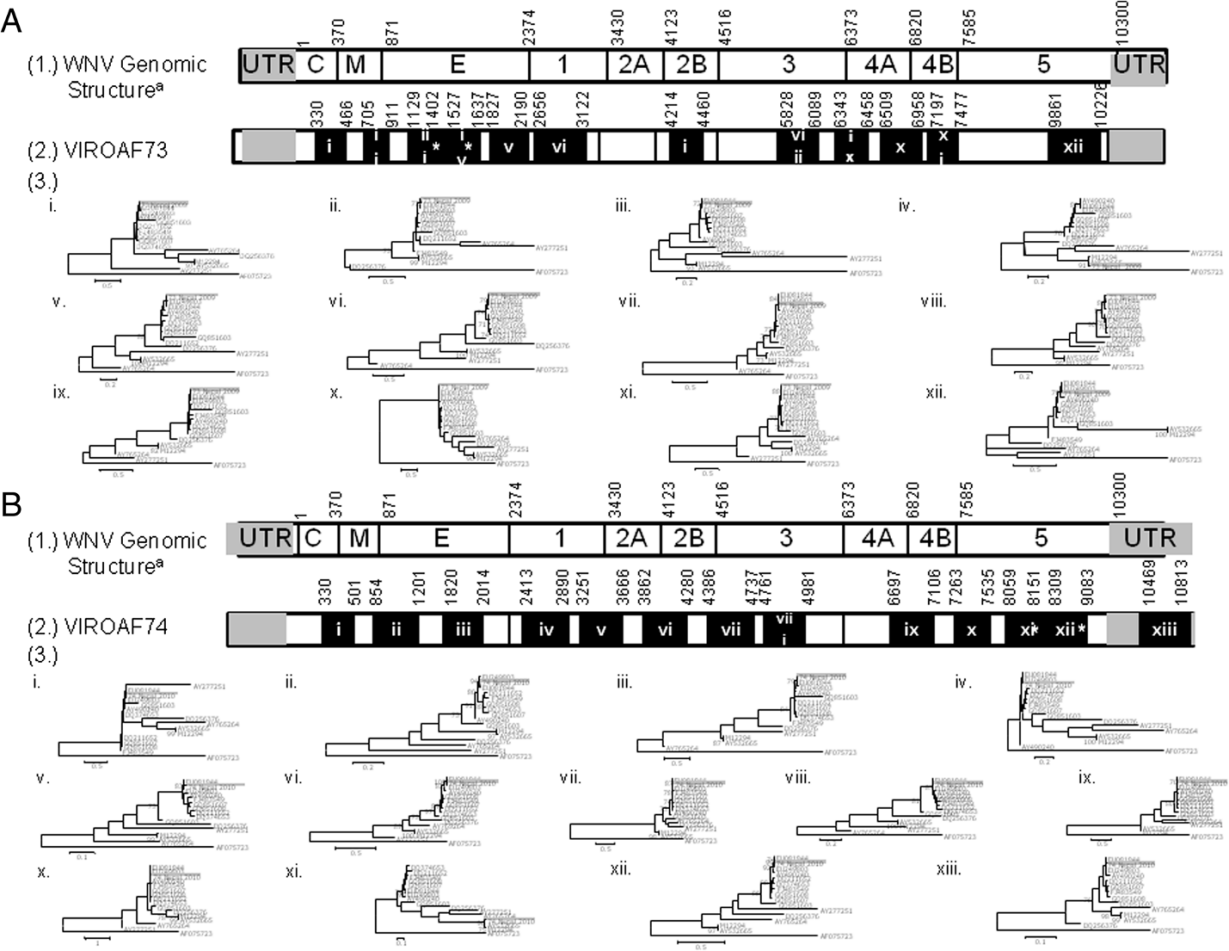
**Genomic structure and Phylogenetic Analysis of Contigs of VIROAF73 (A) and VIROAF74 (B): (1.) Genomic structure of the WNV lineage 1 EU249803 was used as standard for genomic map; (2.) Fragment sizes and genomic locations of contigs from VIROAF73: total of 12 contigs size 207-523 bp and from VIROAF74: total of 13 contigs size 221-942 bp as indicated by number boxes.** (3.) Maximum likelihood phylogenetic trees of fragments of WNV utilizing GTR + G + I model with 15 reference WNV strains and closely matched on GENBANK with denoted WNV lineage and clusters as published by May et al., 2011 (18) and Bondre et al., 2007 (10) (EU249803: India 1968 and EU081844: Egypt 1951 for Clade 1a Cluster1; FJ483549: Italy 2008 for Clade 1a Cluster2; DQ374653: Russia 2002 for Clade 1a Cluster3; DQ211652: NY1999 for Clade 1a Cluster 4; GQ851607: Nigeria 1965 strain for Clade 1a Cluster 5; GQ851608: Central Africa 1967 strain for Clade 1a cluster 6; AY490240: China 2001 and GQ851603: Australia 1991 for Clade 1b; DQ256376: India 1980 for Clade 1c; M12294 and AY532665: Uganda 1937 for Clade 2; AY765264: Czech 1997 for Clade 3; AY277251: Russia 1998 for Clade 4 and AF075723: Japanese Encephalitis India). The scale of genetic distance equal to 0.1-1.0 is indicated in the bottom left of the panel. Brackets designate lineages of WNV on the right side of the tree. Bootstrap equal or greater than 70 is demonstrated next to the node of the tree. ^a^start position of the gene according to the reference KC601756. Asterisk indicates the overlap regions of 126 nt observed between the 3^rd^ fragment and 4^th^ fragment of VIROAF73 and 158 nt observed between the 11^th^ fragment and 12^th^ fragment of VIROAF74. Drawing is not to scale.

RNA from WNV serum was extracted followed by conventional nested RT-PCR. Sample preparation for deep sequencing using MiSeq followed as previously described [9], with an additional centrifugation step at 6,200 g for 10 minutes at 4°C, and DNaseI (PreAnalytiX, QIAGEN, Franklin Lakes, USA) treatment at 37°C for 15 minutes. Sequence reads with at least Q30 score were trimmed to remove adaptor sequences and analyzed as described in [10]. WNV sequence was identified by *de novo* assembly with Trinity followed by Blast or read-mapping align with WNV references (Figure 2). WNV references used in the alignment analysis and maximum likelihood phylogenetic tree were retrieved from GenBank as described, (Figure 2) and the pair-wise genetic distance was calculated using reference strains from lineage 1a and 2 (Table 1). Our sequences were submitted to GENBANK: KJ599821-2 for lineage 1 and 2 for VIROAF73 and KJ599823-4 for lineage 1 and 2 for VIROAF74, respectively.

**Table 1:** **Genetic distance of the WNV sequence fragments from the lineage 1a and 2 reference strains**

Specimen ID.	Fragment ID.	Fragment length (bp)	Genetic distance (p - distance)┼			
			Lineage 1a		Lineage 2	
			EU081844 Egypt 1951	EU249803 India 1968	M12294 Uganda 1937	AY532665 Uganda 1937
VIROAF73	i.	297	0.000	0.000	0.226	0.0234
	ii.	207	0.000	0.000	0.189	0.184
	iii.	400	0.003	0.003	0.229	0.229
	iv.^a^	236	0.242	0.242	0.013	0.004
	v.	364	**0.000**	**0.008**	0.206	0.206
	vi.	467	0.000	0.000	0.221	0.218
	vii.	270	0.056	0.056	0.211	0.219
	viii.	262	0.011	0.011	0.229	0.229
	ix.	208	0.000	0.000	0.216	0.207
	x.	450	0.007	0.007	0.258	0.258
	xi	281	0.000	0.000	0.224	0.224
	xii.	523	0.048	0.048	0.225	0.225
VIROAF74	i.	270	0.000	0.000	0.233	0.238
	ii.	401	**0.083**	**0.085**	0.261	0.261
	iii.	252	**0.129**	**0.133**	0.325	0.325
	iv.	492	**0.018**	**0.020**	0.226	0.226
	v.	473	0.010	0.010	0.185	0.185
	vi	419	0.005	0.005	0.232	0.234
	vii.	352	0.006	0.006	0.179	0.179
	viii.	221	0.000	0.000	0.213	0.213
	ix.	410	0.002	0.002	0.242	0.24
	x.	376	0.015	0.015	0.249	0.249
	xi.^a^	252	0.239	0.239	0.016	0.008
	xii.	942	**0.007**	**0.008**	0.203	0.203
	xiii.	345	0.006	0.006	0.109	0.106

┼Closely related sequence to VIROAF73 & VIROAF74 by blastn on GENBANK: Lineage 1a Reference Strains: Egypt 1951 (EU081844) and India 1968 (EU249803) and Lineage 2 Uganda 1937 (M12294 and AY532665).

^a^Fragments identified to be closely related to Lineage 2.

Numbers in bold indicate the differences of genetic distance from the Egypt 1951 (EU081844) and India 1968 (EU249803).

## Results and discussion

Of 2,046 patients with acute febrile illness without neurological symptoms, 14 had convalescent blood samples positive for WNV antibody by ELISA with most (as there was one DEN positive) excluded for DEN and JE. Only 2 out of 14 samples, identified as VIROAF73 and VIROAF74, showed the presence of WNV RNA by nested RT-PCR with product bands migrating around 104-105 bp as shown by the positive control, WNV Egypt 1951 strain (EU081844). The VIROAF74 104-105 bp amplicon was sequenced to confirm the presence of WNV.

VIROAF73 was obtained from a young adult male student who presented five days after onset of illness in Kathmandu. The temperature, pulse, blood pressure and respiratory rate were measured as 38°C, 100/minute pulse, 100/70 and 22/minute, respectively. His symptoms included anorexia, chills, fever, headache, malaise and muscle aches. The physical examination was remarkable only for abdominal tenderness. There were no reported tick bites, animal exposures or contacts with a person with similar symptoms. On presentation, he had been taking paracetamol and Ayurvedic for 5 days. He reported no significant past medical history. He lived in the urban area of Kathmandu and had no recent travel history.

VIROAF74 was obtained from a middle-aged male in the service industry who was hospitalized in Bharatpur seven days after onset of illness. His symptoms included abdominal cramps, fatigue, fever, headache and malaise with joint and retro-orbital pain. His temperature, pulse, blood pressure and respiratory rate were 40.3°C, 100/minute, 100/70 and 20/minute. The physical examination indicated that he was pale and lethargic with no obvious tick bites. He had exposure to cattle and buffalo and had contact with a household member with similar symptoms. He had not traveled outside of Bharatpur, which is an urban center near the border with India. His treatment at time of presentation included Cefixime 200 mg tablet BD for 5 days. He had no significant past medical history. He was also found to have a potential acute secondary dengue infection based on elevated dengue IgM and IgG antibodies with IgM: IgG ratio <1.8 which remained elevated, although slightly decreased, in his convalescent specimen. This could represent a co-infection or cross-reactive elevation of pre-existing dengue antibodies upon immune boosting with another flavivirus.

**Figure 3:**
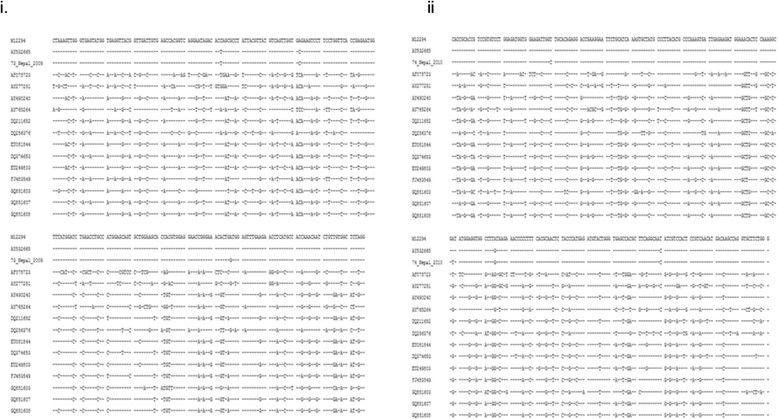
**Alignment of WNV lineages comparing fragment 4 of VIROAF73 (A) and fragment 11 (B) of VIROAF74 against 4 WNV lineages.** For the alignment, the dashed line indicates the presence of the same base as the master template WNV lineage 2, which classifies these fragments as WNV lineage 2 phylogenetically (Figure 2).

Deep sequencing was conducted on both VIROAF73 and VIROAF74 sera. Following the pathogen identification algorithm, both read-mapping alignment and *de novo* assembly procedures identified several fragments aligning with the WNV genome. Overall, approximately 36% of the WNV genomic sequence was found in VIROAF73 and 44% in VIROAF74 (Figure 2). A total of 1306 bp covered over nine overlapping regions between VIROAF73 and VIROAF74, which had 97.0%-100.0% nucleotide identity. For VIROAF73, a total of 12 contig fragments with sizes ranging from 116 to 450 bp were mapped from the 5’ end to the 3’ end of the WNV genomes. Eleven of 12 contig fragments fell in the same group of WNV lineage 1a (Figure 2A and Table 1). Similarly, for VIROAF74, 12 out of 13 contig fragments with sizes ranging from 172 to 933 bp were grouped with WNV lineage 1a (Figure 2B and Table 1). The sequences of these VIROAF73 and VIROAF74 fragments were slightly different from the lineage 1a reference strains (Table 1), which had nine regions of 1306 bp in length overlap with an average of 0.007 (0.000 – 0.030) pair-wise distance between them. Contig 4 of VIROAF73 aligned with an envelope region of WNV which shared more similarity with lineage 2 of WNV than lineage 1 (Figure 3). The same was observed with contig 11 of VIROAF74 which aligned with the NS5 gene of WNV (Figure 3).

## Conclusions

Our study identified two cases of WNV infection by advanced molecular and 14 probable cases by serological techniques from a study that enrolled 2,046 febrile illness patients in Nepal from May 2009 to December 2010. Initial evidence of WNV in the specimens was provided by nested PCR amplicons co-migrating with the WNV positive control. Further analyses of the sequences obtained using deep sequencing corroborated the presence of WNV.

In both patients, the convalescent sera also contained WNV antibodies as determined by Panbio ^(R)^ West Nile Virus IgM Capture ELISA, suggesting the clinical specimens were collected during the declining stage of WNV viremia [11]. Despite incomplete WNV sequence recovered by deep sequencing, possibly due to low WNV viremia in the clinical specimens, fragment sequences obtained from VIROAF73 and VIROAF74 are likely WNV lineage 1 as determined by *de novo* assembly and alignment with WNV Egypt strain 101. Using Blastn and GENBANK sequences we found that almost all contigs (11/12 fragments of VIROAF73 and 12/13 fragments of VIROAF74) were closely related to WNV lineage 1a cluster 1 from India (strain 68856) and from Egypt (strain 101) and less so to strain China 2001 [2].

Nonetheless, the presence of sequences obtained from VIROAF73 (envelope) and from VIROAF74 (NS5B), which aligned with WNV lineage 2 (Figure 3), suggests that the WNV in the two specimens might be of a distinct strain since WNV lineage 2 is usually found in sub-Saharan Africa and Madagascar. WNV lineage 2 isolates have also been identified in South Africa [12], Hungary [13], and Russia [2]. The detection of a small portion of lineage 2 in lineage 1a backbone in VIROAF73 and VIROAF74 in Nepal suggests the presence of two lineages of WNV in both specimens. The detection of more than two lineages of WNV has been previously reported [14]-[16]. This may be associated with introduction by migratory birds as has been suggested in other settings [2].

The presence of WNV in two acute serum samples collected from Kathmandu and Bharatpur demonstrates the expansion of the virus to Nepal. Similarly, other vector borne diseases have recently been detected in Nepal for the first time, such as dengue virus, first detected in 2008, although much of Nepal is at a higher altitude than other endemic areas [17]. The detection of these distinct WNV strains from individuals without neurological symptoms represents the identification of a new etiology for acute febrile illness in Nepal. In a country endemic for another flavivirus, Japanese encephalitis, it can also be useful to consider WNV infection with acute central nervous system infection.

### Consents

Written informed consent was obtained from the patient for participation in the febrile illness study. A copy of the written consent is available for review by the Editor of this journal.

## Authors’ original submitted files for images

Below are the links to the authors’ original submitted files for images. Authors’ original file for figure 1 Authors’ original file for figure 2 Authors’ original file for figure 3 Authors’ original file for figure 4 Authors’ original file for figure 5
