# Supplementary figures and images for: Evidence of West Nile virus infection in Nepal
Source: BMC Infect Dis. 2014 Nov 27;14:606. doi: 10.1186/s12879-014-0606-0 (PMC4265323; doi:10.1186/s12879-014-0606-0)

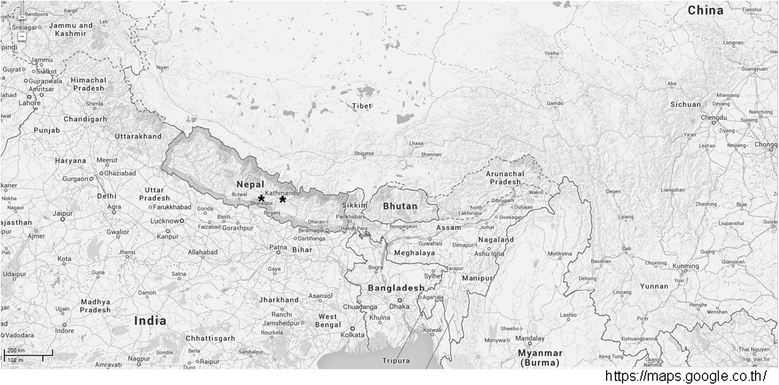

Supplement: Supplementary file 1 — Authors’ original file for figure 1 [file 12879_2014_606_MOESM1_ESM.gif]

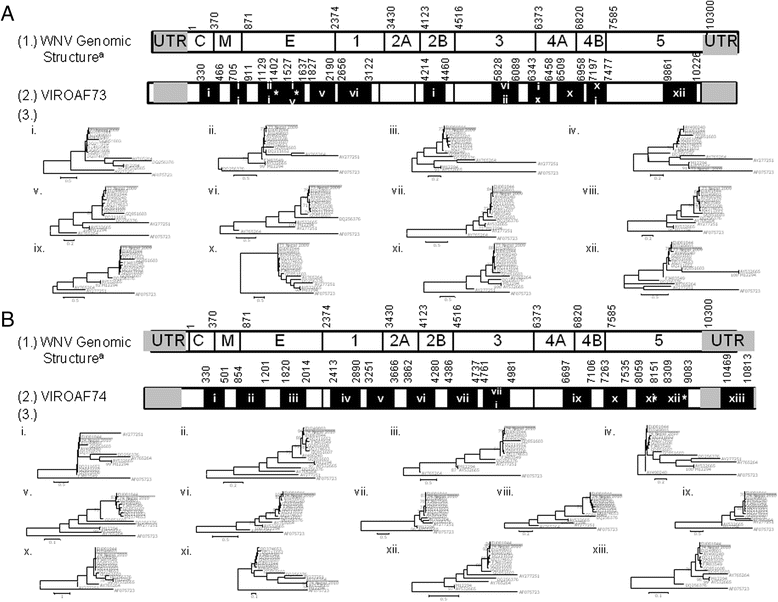

Supplement: Supplementary file 2 — Authors’ original file for figure 2 [file 12879_2014_606_MOESM2_ESM.gif]

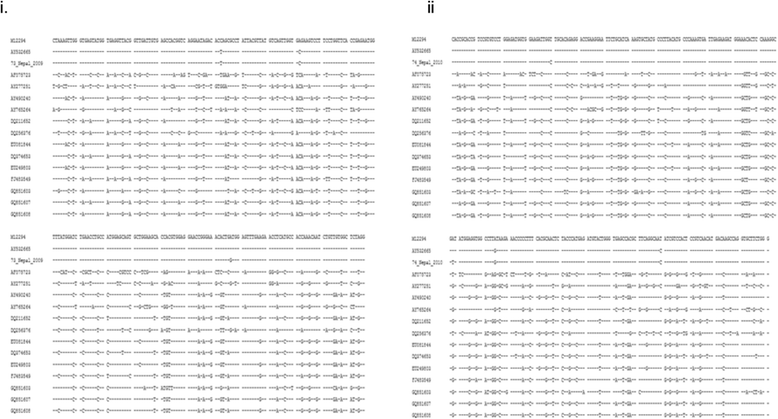

Supplement: Supplementary file 3 — Authors’ original file for figure 3 [file 12879_2014_606_MOESM3_ESM.gif]
