# Supplementary material for: Evidence of West Nile virus infection in Nepal
Source: BMC Infect Dis. 2014 Nov 27;14:606. doi: 10.1186/s12879-014-0606-0 (PMC4265323; doi:10.1186/s12879-014-0606-0)
Supplement: Supplementary file 4 — Authors’ original file for figure 4 [file 12879_2014_606_MOESM4_ESM.ppt]

## Slide 1
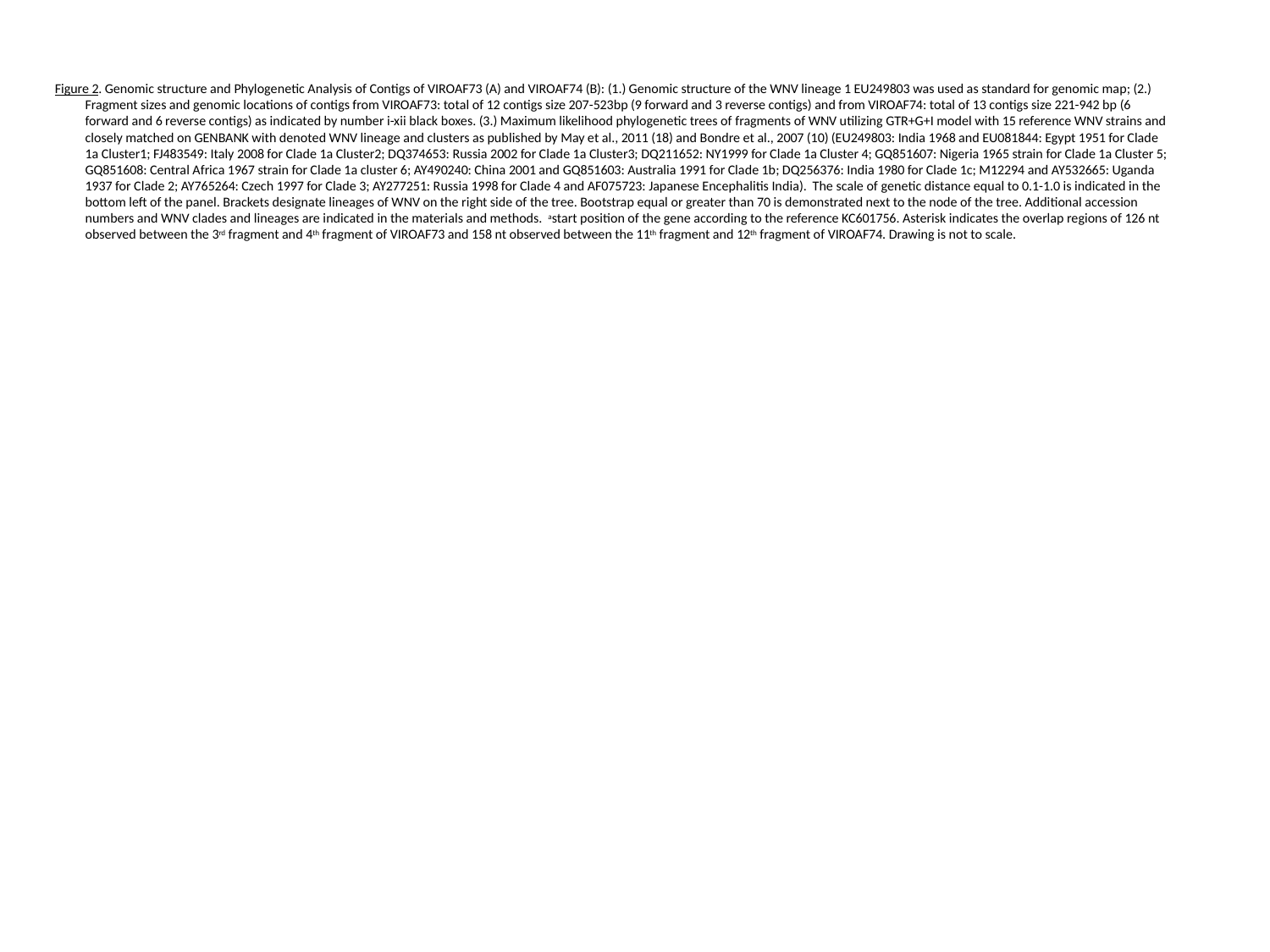

Figure 2. Genomic structure and Phylogenetic Analysis of Contigs of VIROAF73 (A) and VIROAF74 (B): (1.) Genomic structure of the WNV lineage 1 EU249803 was used as standard for genomic map; (2.) Fragment sizes and genomic locations of contigs from VIROAF73: total of 12 contigs size 207-523bp (9 forward and 3 reverse contigs) and from VIROAF74: total of 13 contigs size 221-942 bp (6 forward and 6 reverse contigs) as indicated by number i-xii black boxes. (3.) Maximum likelihood phylogenetic trees of fragments of WNV utilizing GTR+G+I model with 15 reference WNV strains and closely matched on GENBANK with denoted WNV lineage and clusters as published by May et al., 2011 (18) and Bondre et al., 2007 (10) (EU249803: India 1968 and EU081844: Egypt 1951 for Clade 1a Cluster1; FJ483549: Italy 2008 for Clade 1a Cluster2; DQ374653: Russia 2002 for Clade 1a Cluster3; DQ211652: NY1999 for Clade 1a Cluster 4; GQ851607: Nigeria 1965 strain for Clade 1a Cluster 5; GQ851608: Central Africa 1967 strain for Clade 1a cluster 6; AY490240: China 2001 and GQ851603: Australia 1991 for Clade 1b; DQ256376: India 1980 for Clade 1c; M12294 and AY532665: Uganda 1937 for Clade 2; AY765264: Czech 1997 for Clade 3; AY277251: Russia 1998 for Clade 4 and AF075723: Japanese Encephalitis India). The scale of genetic distance equal to 0.1-1.0 is indicated in the bottom left of the panel. Brackets designate lineages of WNV on the right side of the tree. Bootstrap equal or greater than 70 is demonstrated next to the node of the tree. Additional accession numbers and WNV clades and lineages are indicated in the materials and methods. astart position of the gene according to the reference KC601756. Asterisk indicates the overlap regions of 126 nt observed between the 3rd fragment and 4th fragment of VIROAF73 and 158 nt observed between the 11th fragment and 12th fragment of VIROAF74. Drawing is not to scale.
