# Supplementary material for: Evidence of West Nile virus infection in Nepal
Source: BMC Infect Dis. 2014 Nov 27;14:606. doi: 10.1186/s12879-014-0606-0 (PMC4265323; doi:10.1186/s12879-014-0606-0)
Supplement: Supplementary file 5 — Authors’ original file for figure 5 [file 12879_2014_606_MOESM5_ESM.ppt]

## Slide 1
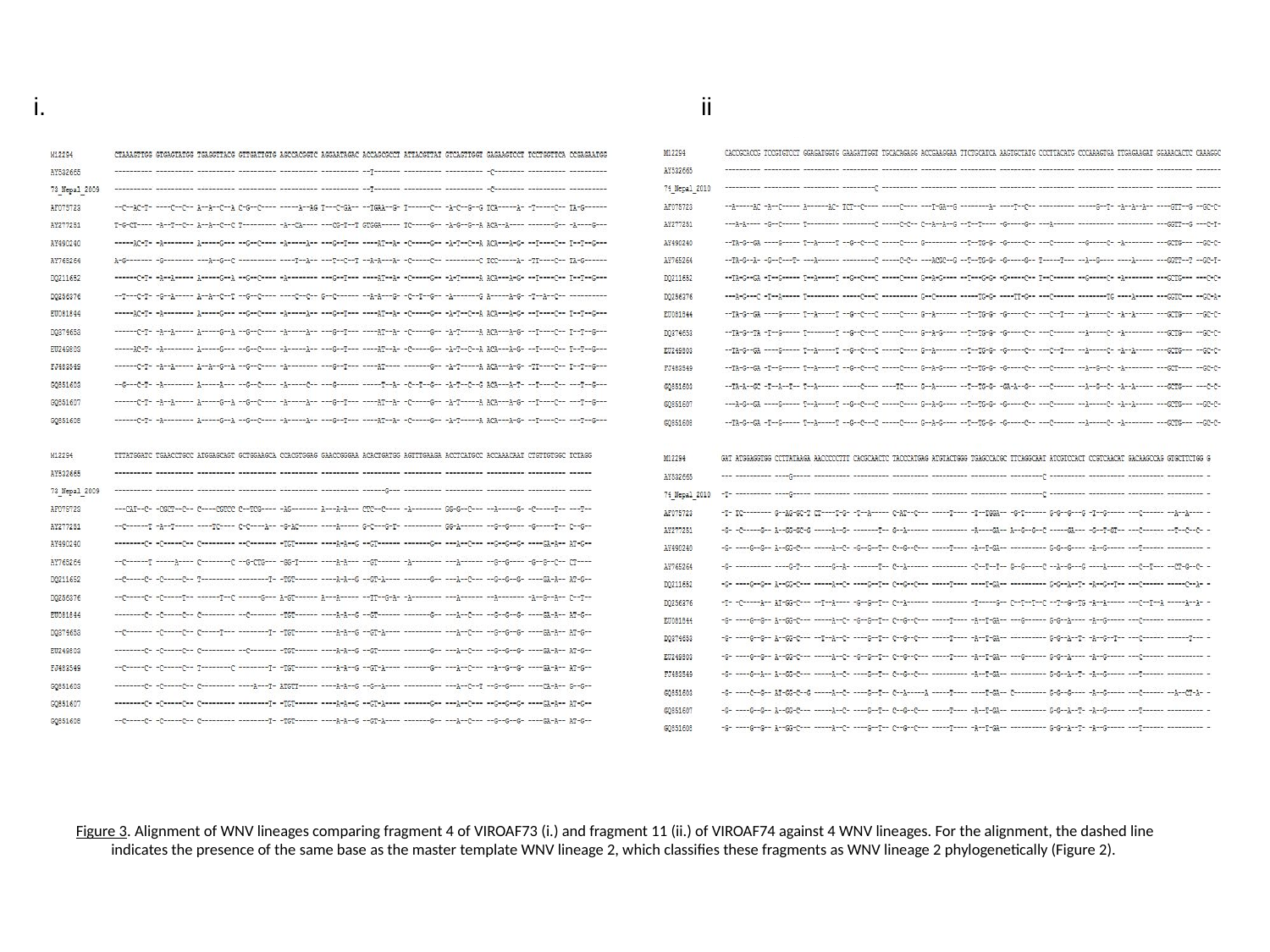

i. ii
Figure 3. Alignment of WNV lineages comparing fragment 4 of VIROAF73 (i.) and fragment 11 (ii.) of VIROAF74 against 4 WNV lineages. For the alignment, the dashed line indicates the presence of the same base as the master template WNV lineage 2, which classifies these fragments as WNV lineage 2 phylogenetically (Figure 2).
